# Impact of Oral Hygiene Practices in Reducing Cardiometabolic Risk, Incidence, and Mortality: A Systematic Review

**DOI:** 10.3390/ijerph21101319

**Published:** 2024-10-04

**Authors:** Lauren Church, Kay Franks, Nidhi Medara, Karolina Curkovic, Baani Singh, Jaimit Mehta, Raied Bhatti, Shalinie King

**Affiliations:** 1 Sydney Dental School, The University of Sydney, Sydney 2006, Australia; 2 Westmead Applied Research Centre, The University of Sydney, Westmead 2145, Australia; 3 School of Health Sciences, Oral Health, The University of Newcastle, Ourimbah 2258, Australia

**Keywords:** cardiometabolic disease, cardiovascular disease, type 2 diabetes mellitus, kidney disease, oral health, toothbrushing

## Abstract

Cardiometabolic diseases share many modifiable risk factors. However, periodontitis, a chronic inflammatory condition of the gums, is a risk factor that is rarely publicized. This systematic review aims to evaluate the impact of oral hygiene practices on the risk, incidence, and/or mortality rate of cardiovascular disease (CVD), type 2 diabetes mellitus (T2DM), and chronic kidney disease (CKD). Searches were conducted using MEDLINE, Embase, Scopus, and CINHAL. Randomized controlled trials (RCTs), quasi-RCTs, and observational studies were included. Eligible studies reported on associations of toothbrushing, interdental cleaning, mouthwash, or toothpaste use, either alone or in combination with CVD, CKD, and/or T2DM outcomes in adults ≥ 18 years. Fifty-five studies were included. Cochrane’s risk of bias tool and the Newcastle–Ottawa Scale were used for quality assessment. Data synthesis is narratively presented. Toothbrushing and interdental cleaning were associated with lower risk of developing T2DM or hypertension HR 0.54 [*p* < 0.001] and a lower mortality risk in those with CVD HR = 0.25 [*p* = 0.03]. Mouthwash use reportedly increased the risk of developing hypertension and diabetes by 85% and 55%, respectively. This review highlights how simple oral hygiene practices can reduce cardiometabolic risk. Non-dental clinicians could integrate the findings into chronic disease health promotion.

## 1. Introduction

Cardiometabolic diseases encompass a group of interrelated conditions including cardiovascular disease (CVD), diabetes mellitus (DM), and chronic kidney disease (CKD) [1], and are increasingly occurring in combination [2]. These conditions are the leading cause of death and disability worldwide [3,4,5], and collectively were responsible for almost half of all Australian deaths in 2022 [6,7,8]. Cardiometabolic diseases share many highly publicised, modifiable risk factors, including tobacco and alcohol consumption, diet, and sedentary lifestyle [6,7,8]. However, oral disease, specifically periodontal disease, (PD) [9] which is another important modifiable risk factor, is seldom mentioned [6,7,8]. Categorised as gingivitis or periodontitis, PD affects 90% of any population [10]. The more severe form of PD, periodontitis, which affects between 20 and 50% of the global population [11], has strong associations with CVD [12,13] and a well-established bi-directional relationship with DM [14]; and there is also emerging evidence of a bi-directional link with CKD [15,16].

Preventing PD and other oral disease [17] begins with simple oral hygiene practices including toothbrushing (TB) with toothpaste, interdental cleaning, and, under certain circumstances, mouthwash use which together maintain a healthy oral biofilm [18,19,20,21]. Poor oral hygiene practices allow bacterial deposits to accumulate on the soft and hard tissues of the oral cavity resulting in PD. A biofilm that remains stagnant for 24–48 h, initiates a host immune response, triggering a local inflammatory reaction which in turn initiates systemic inflammation [22]. The local vasodilation enables bacteria within stagnant biofilm to enter the bloodstream [22] and reach distant organs such as the heart and pancreas [13]. Inflammation is therefore the mechanism that links the more severe form of PD, namely periodontitis, and cardiometabolic conditions such as CVD, CKD, and type 2 diabetes mellitus (T2DM) [13,23]. Whilst previous studies investigated oral hygiene practices in combination with other therapies [24], or focus on a single cardiometabolic condition [25], this study is the first systematic review to evaluate the effect oral hygiene practices alone may have on a range of cardiometabolic conditions. The aim of this review is to identify the impact of oral hygiene practices on the risk, incidence, and/or mortality rate of CVD, T2DM, and CKD.

## 2. Materials and Methods

This systematic review was conducted in accordance with the Preferred Reporting Items for Systematic Reviews and Meta-Analysis (PRISMA) statement [26]. The review protocol is registered with an international register of systematic reviews (PROSPERO ID: CRD42021269584).

### 2.1. Inclusion and Exclusion Criteria

Peer-reviewed randomized controlled trials (RCTs), quasi-RCTs, observational, cohort, case–control, and cross-sectional studies were eligible for inclusion meeting the following criteria: adults ≥18 years, examined the influence of oral hygiene practices (toothbrushing, interdental cleaning, mouthwash, or toothpaste use, either alone or in combination) on cardiometabolic disease, specifically CVD, CKD, and/or T2DM. Conference abstracts, case-studies/series, letters to the editors, editorials, and animal studies were excluded. Studies which reviewed scaling and root debridement/planning, periodontal surgery, or other dental treatments alone, or focused on other cardiometabolic conditions or non-cardiometabolic diseases were excluded.

### 2.2. Search Strategy

The initial search was performed on 27 July 2021 and repeated in 2022 and 2023. The final search, occurring on 10 July 2024, was carried out on the following electronic databases: MEDLINE, Embase, Scopus, and CINHAL. No restrictions were placed on language or publication period, and no human filter was applied. Mirroring terms were used for electronic databases; Google Scholar was explored for grey literature. Citation lists of relevant studies were also examined. The search strategy included Boolean operators, medical subject headings, and truncations, and included terms relating to, or describing, oral health practices and cardiometabolic diseases, including CVD, T2DM, and CKD. Every attempt was made to retrieve inaccessible studies. For the full search strategy, see Supplementary Material S1.

### 2.3. Data Extraction and Synthesis

A data extraction tool developed within Covidence was completed independently by LC and one of five other reviewers (BS, KC, KF, JM, RB). Key characteristics extracted were author[s], publication year, country, study design, aim[s], setting, age, sex, oral hygiene practices, disease biomarkers, and oral health status. Due to the heterogeneity in included study designs and outcomes, a meta-analysis was not feasible. Data synthesis has been presented narratively with reference to Supporting Material.

### 2.4. Quality Appraisal and Risk of Bias

Study quality was assessed independently by LC and one of five other reviewers (BS, KC, KF, JM, RB). RCT quality (n = 3) was assessed using an adapted Cochrane’s risk of bias tool [27]. The Newcastle–Ottawa Scale (NOS) [28] was used for all other included studies (n = 52). Any disagreements were resolved via open discussion and where consensus was unsuccessful a third investigator (SK or NM) was involved.

## 3. Results

### 3.1. Selection Process

**Figure 1:**
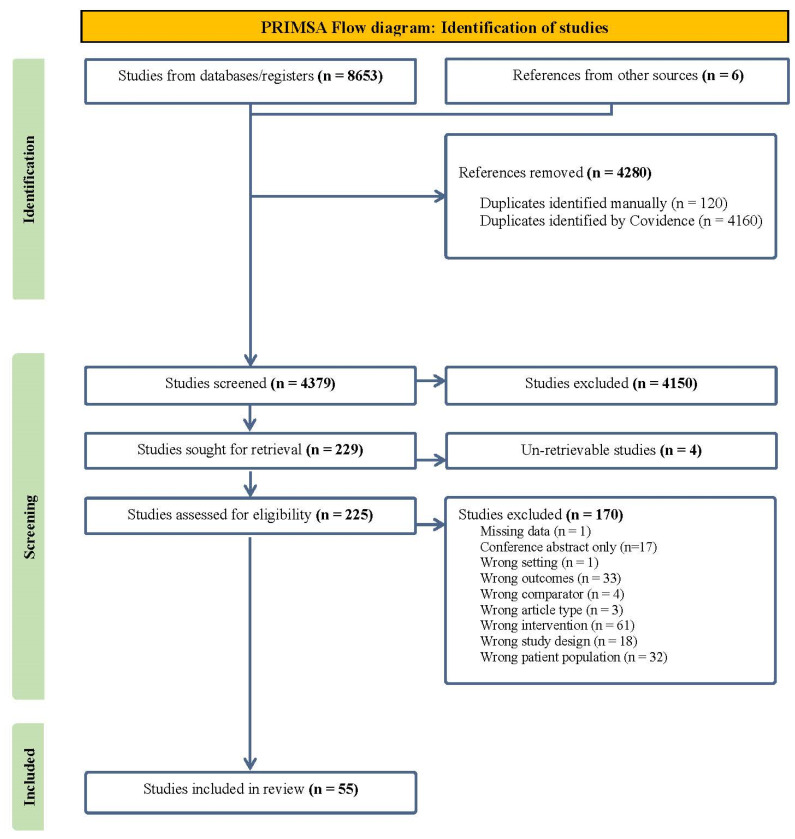
PRISMA Flow diagram.

Using systematic review software Covidence [29], 8659 citations were imported into the program, 4280 duplicates were removed and 4379 were screened. Title and abstract screening, followed by full-text screening, was completed independently by two reviewers from a pool of eight (BS, KC, KF, JM, LC, NM, RB, SK). A total of 55 studies (32 cohort [30,31,32,33,34,35,36,37,38,39,40,41,42,43,44,45,46,47,48,49,50,51,52,53,54,55,56,57,58,59,60,61], 13 cross-sectional studies [62,63,64,65,66,67,68,69,70,71,72,73,74,75], 3 case–control [76,77,78], 1 prospective [79], 2 observational [80,81], 1 quasi-experimental [82], and 3 RCTs [83,84]) met the inclusion criteria. See Figure 1 for the PRISMA flow diagram. Conflicts arising during the screening process were resolved via group discussion.

### 3.2. Characteristics

Fifty-five studies spanning between 2003 and 2024 were identified, taking place in Europe [45,46,50,65,76], Asia [31,32,34,36,39,40,43,44,47,48,49,52,53,54,55,56,57,58,59,60,61,62,63,67,68,69,70,71,72,73,77,78,81,82,84], United States [30,51,66,74,75], South America [33,38,41,42,45], and the Middle East [35,64,79,80,83]. Study populations ranged from n = 60 to n = 487,198; the mean and median age ranges were 41.5 ± 9.3–67.71 ± 10.6 and 63–68, respectively. Eleven studies had a majority of female participants, twenty-eight had a majority of male participants, two included only male participants and four did not disclose sex. Thirty-four studies focused on CVD [31,36,37,40,42,43,44,46,47,49,51,52,53,54,55,56,57,59,60,61,62,65,66,67,68,69,71,72,73,76,77,78,81], twenty-three on DM [30,32,34,35,41,43,47,48,49,50,53,57,63,64,67,68,69,70,72,74,75,79,82,83,84], and nine on CKD [33,38,39,45,49,58,69,80,81]. Oral hygiene practices investigated included TB [31,32,33,34,35,36,37,39,40,43,44,45,46,48,49,50,51,52,53,54,55,56,57,58,59,60,61,62,63,63,64,65,66,67,68,69,70,72,73,74,76,77,77,78,78,79,80,81,82,83,84], interdental cleaning [30,33,34,38,45,46,51,62,66,71,74,75,76,81,82], mouthwash [33,41,42,45,51,62,76,78,79,81,82,84], and fluoridated toothpaste use [34,77,78]. See Table 1 for study characteristics.

Two studies reported oral hygiene practices as a proxy for oral hygiene status score [51,81], thus not directly relating specific practices with the disease outcome. Additionally, two discussed the relationship of oral hygiene practice to oral conditions (non-defined periodontal symptoms and tooth loss) [63,71], which were associated with the disease outcome; see Table 2 for study findings.

Of the total studies, thirty-nine [30,31,33,36,37,38,39,40,41,42,43,46,48,49,50,52,53,54,55,56,57,58,59,60,61,62,65,66,67,68,71,72,73,74,75,76,77,78,80] were considered good quality, eight [37,45,47,48,63,69,70,81] were considered fair, and the remaining five [34,35,51,64,79] were rated poor quality; see Table 3 for NOS quality assessment.

**Figure 2:**
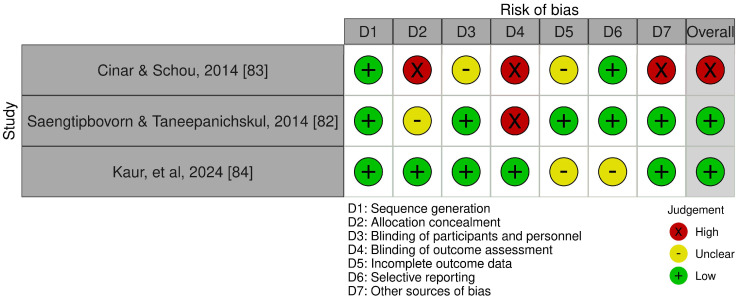
RCT risk of bias.

The RCTs’ overall risk of bias was determined high [n = 1] [83] and low [n = 2] [82,84]; see RoB in Figure 2. Created using the robvis tool with permission [85].

### 3.3. Toothbrushing Frequency

Forty-three studies examined TB practices reported as daily frequency [31,32,33,34,35,36,37,39,40,43,44,45,46,47,48,49,50,51,52,53,55,56,57,58,59,60,61,62,63,64,65,66,67,68,69,72,73,74,76,77,78,79,80,81,82,83], device used [51,77,78,84], technique [63,77], time of day [54,70,77], and/or duration [45,55]. Increased TB frequency (ranging from sometimes/daily—≥3× daily) was linked to reduced risk of developing CVD [68,78,81] and/or hypertension. [31,53,56,67] It was also shown to reduce the risk of stroke [36,40,52,59,62,77], atrial fibrillation [37], and/or heart failure (HF) occurrence [37,57], cardiovascular events [40,44,49,51,52,55,65,74], including death [51,60,61,73,76], and all-cause deaths [49,60]. Seven studies reported no link between low TB frequency (ranging from never to 2× daily) and increased risk of hypertension [43], acute coronary syndrome [40], cardiovascular death [45], subarachnoid haemorrhage, ischemic heart disease [49,60], haemorrhagic stroke [59], or other CVD outcomes [46].

For individuals living with DM, higher daily TB frequency was found to significantly lower the risk of cerebral or myocardial infarction [47] as well as HF [57]. One study found lower daily TB frequency was a risk factor for developing DM in males, but not females [43], whilst two discussed no relationship between TB frequency and T2DM [49,74]. Several studies highlighted either increased DM prevalence associated with lower TB frequency [67,69,72] or reduced DM incidence associated with higher TB frequency [31,32,50,53]. Similarly, in those living with end-stage kidney disease (ESKD), individuals with higher TB frequency had lower prevalence of hypertension, DM [39], rates of hospitalisations [33], and longer all-cause survival [45]; however, there was no impact on cardiovascular death risk.

Conversely, one study reported that TB frequency was most the important factor for all-cause peritonitis, streptococcal peritonitis, CHF, and pneumonia in individuals with ESKD [81]. Two [49,69] found no association between TB frequency and CKD, whilst another reported an inverse relationship between TB frequency and developing CKD [58]. Interestingly, one study concluded TB only 1–2× daily was associated with better kidney function [39].

Twenty studies reported on biomarkers linked to CVD, DM, and CKD. These included C-reactive protein (CRP) or high-sensitivity C-reactive protein (hs-CRP), interleukin (IL), blood glucose, cholesterol, lipids, and others. Seven studies reported a significant inverse relationship between TB frequency and circulating CRP/hs-CRP [55,65,73,73,76,80,81].

One study linked TB frequency with undefined periodontal symptoms, which were found to be associated with higher glycated haemoglobin (HbA1c) levels [63]. The other studies reported lower Hb1Ac [35,82,83], fasting plasma glucose (FPG) [82], or fasting blood glucose (FBGL) [48,73] with more frequent TB. One study concluded those who did not brush daily had a 79% increased risk of higher HbA1c [79], similar to another study [34].

Biomarkers related to dyslipidaemia are heavily involved with cardiometabolic conditions and were discussed in seven studies [43,64,66,67,69,73,79]. Whilst one study reported total cholesterol was not significantly impacted by TB frequency [67], others found that less frequent TB was associated with elevated total cholesterol [66], lower HDL [64,67,73,79], high LDL [79], and high triglycerides [67,73]. Two studies found reduced TB frequency was associated with dyslipidaemia [43,69]. Interestingly; however, one found frequency was linked to developing the condition in females only [43].

The impact of TB frequency on other cardiometabolic disease biomarkers had similar results. Less frequent TB was associated with increased concentrations of white blood cells [73], adiponectin [66], fibrinogen, higher levels of IL-1β, and a trend toward an association with a higher level of tissue plasminogen activator inhibitor-1 (tPAI-1) [65]. A better oral hygiene score was significantly associated with lower albumin levels [81] and had an inverse correlation with oral innate immunity marker salivary lysozyme [76]. Additionally, increased TB frequency was associated with higher estimated glomerular filtration rate [eGFR] and milder stage of CKD compared to less frequent TB [39].

### 3.4. Toothbrushing Device

Three studies examined the impacts of TB devices on CVD, whilst one focused on T2DM. One study found, compared to controls, participants living with stroke were less likely to use a toothbrush, leading to poor oral health status and increased periodontal disease [77]. Another reported that the use of other toothbrushing devices such as a neem stick or miswak increased the risk of developing CHD [78], whilst using an electric toothbrush was found to be associated with a decrease in non-specified CVD outcomes and better survival when combined with other oral hygiene practices [51]. When comparing electric and manual tooth brushes, one study found significant reduction in HbA1c in both study arms at the final follow-up; however, no significant reductions in CRP and IL-β levels were found [84].

### 3.5. Toothbrushing Technique

Toothbrushing technique (non-rolling or rolling stroke) had no significant effect on self-reported periodontal symptoms [63] and no significant differences were detected between individuals living with or without stroke in terms of their toothbrushing technique (horizontal or other) [77].

### 3.6. Timing

The timing of toothbrushing was assessed in three studies. When defined as before or after meals, one study found no association with toothbrush timing and individuals living with stroke, compared to those without stroke [77]. Defined as brushing before bed (sometimes/no or always) in a T2DM cohort, those who brushed their teeth every night before bed had lower BMI and LDL than those who did not brush nightly [70]. The third study categorised TB timing as Group MN (morning and night)/Group M (morning only)/Group Night (night only)/Group None (not brushing) and reported higher survival estimates following cardiovascular events in Group MN and Group N [54].

### 3.7. Duration

The duration of TB was assessed in two studies. In individuals living with ESKD, there was an association between longer survival and lower risk of cardiovascular death when TB for ≥2 min [45]. For individuals living with CVD, brushing < 2× daily for <2 min was associated higher risk of cardiovascular events compared to those TB for ≥2 min ≥2× daily [55]. However, no differences were detected for cardiovascular events when comparing brushing < 2× daily for <2× min with brushing either twice daily or for two minutes.

### 3.8. Interdental Cleaning

Interdental cleaning was evaluated in fourteen studies. As the primary focus of [30,38,75], dental floss use was associated with lower HbA1c levels [75] and lower risk of elevated CRP [30], and its lack of use was associated with an increased risk for patient mortality [38]. As a proxy for remaining teeth, seldom/occasional dental floss use before bed was indirectly linked to higher risk of stroke [71]. Secondary to other oral hygiene practices, dental floss or interdental brush use was associated with reduced hospitalisations [33], decreased risk of CVD mortality [45,46,51,76], increased adiponectin and fibrinogen levels [54], and longer survival rates if living with ESKD [45]. In contrast, less-frequent flossing was associated with elevated mean arterial pressure. Increased daily use of dental floss in a diabetic population was indirectly linked to the decrease in FPG and HbA1c [82].

Furthermore, the use of dental floss was linked to health outcomes when grouped with other practices to develop an oral hygiene score [51,81]. For individuals living with CKD completing peritoneal dialysis, an oral hygiene score of ≥7 was associated with lower hospitalisation and mortality rates [81]. One study reported individuals who had a high oral hygiene score had a 79% decreased risk of CVD morality [51]. Similarly, when grouped with other dental products, one study reported an association with interdental cleaning and stroke [62].

### 3.9. Mouthwash and Toothpaste

Daily mouth wash use was discussed in twelve studies. Three incorporated the practice with either auxiliary products [62] or other practices [81,82], indirectly linking its use to improved glycaemic control [82], reduced CVD admissions, and mortality rates [81], as well as an association with stroke [62]. Significant reductions in H1bAc levels at follow-up were reported by one study when using mouthwash via a water flosser and a powered brush twice daily [84]; however, no significant reductions were reported in CRP or IL-β. Two studies found mouthwash use corresponded with better survival [45] and lower hospitalisations [33] in individuals living with ESKD and CKD. Two studies [41,42] found that its use was associated with a 55% and 85% higher risk of developing new-onset DM or hypertension, respectively, as well as increased blood pressure [42]. However, other studies found no independent link between mouthwash use and CVD outcomes [51,76]. One study included both mouthwash and toothpaste use, though it did not link them to HbA1c [34]. Two [77,78] found no independent link with toothpaste use and disease outcomes.

## 4. Discussion

This systematic review is the first to assess the impact of oral hygiene practices on the risk, incidence, and/or mortality rate of cardiometabolic conditions CVD, T2DM, and CKD, and highlights the significant effects some of these practices have on cardiometabolic disease outcomes.

### 4.1. Main Findings

Frequency of TB was the primary focus of most studies [31,32,33,34,35,36,37,39,40,43,44,45,46,47,48,49,50,51,52,53,55,56,57,58,59,60,61,62,63,64,65,66,67,68,69,72,73,74,76,77,78,79,80,81,82,83]. Although there were some conflicting results [40,43,45,46,49], the overwhelming consensus was that increased TB frequency had positive impacts on CVD [31,36,37,40,43,44,47,49,52,53,54,55,56,57,59,60,61,62,65,66,67,68,69,72,73,77,78], DM [30,31,32,34,35,43,47,48,49,50,53,57,63,64,67,69,72,74,79,82,83], and CKD [33,39,45,49,58,69,80,81] outcomes. These findings support the role oral bacteria play in cardiometabolic disease if allowed to remain stagnant. Furthermore, the combination of increased TB frequency and daily interdental cleaning was associated with a further decrease in CVD mortality risk compared to brushing alone [76]. This result is likely due to interdental cleaning reaching areas where toothbrushes cannot [20].

Increased frequency of oral hygiene practices was also found to reduce inflammatory markers such as CRP [30,55,65,66,73,76,80,81], further supporting the role of oral bacteria in driving systemic inflammation. Elevation of CRP levels are associated with the development of cardiometabolic conditions [86,87], having detrimental consequences for those already living with CVD [87], DM [86], and CKD [88].

Interestingly, for individuals living with CKD, there were contradictory findings. Two studies found TB ≥ 3 times daily had positive CKD outcomes [33,58], whilst another reported TB after every meal was more detrimental to kidney function than TB 1–2× daily [39]. This may be due to disruption of the oral microbiome as overbrushing has been reported to reduce beneficial bacteria [89]. The other studies involving CKD do not define TB frequency [45] or incorporate it into an oral hygiene score [80,81], and as such, additional studies with standardised TB frequency categories are required to clarify these opposing findings.

The benefits of mouthwash use on cardiometabolic disease outcomes is inconclusive with studies reporting positive [33,45,81,84], neutral [51,76], and negative [41,42] health outcomes. The indeterminate bactericidal nature of mouthwash which can disrupt the healthy oral microbiome is thought to be the reason for the negative health outcomes.

The oral microbiome is a complex mix of beneficial bacteria and other microorganisms residing in the oral cavity [90]. This microbiome, as well as protective enzymes in saliva [91], make up part of a nonimmune defence system against invasion from harmful bacteria and pathogens [90,91]. Mouthwash use disrupts the oral microbiome by eliminating both beneficial and harmful bacteria [92] and may explain why ≥2× daily use was found to increase the risk of hypertension and DM [41,42].

### 4.2. Risk Factor Comparison

Diet and exercise are well-known modifiable risk factors for cardiometabolic diseases [6,7,8]. Recent literature found individuals with a higher diet score (≥5) were at a lower risk of mortality (HR 0.70;0.63–0.77), CVD (HR 0.82; 0.75–0.91), MI (HR 0.86; 0.75–0.99) and stroke (HR 0.81; 0.71–0.93) [93]; having a combination of a healthy diet and active lifestyle significantly lowered the risk of all-cause mortality (HR 0.74; 0.65–0.86) and CVD deaths (HR 0.79; 0.73 to 0.86) [94].

Similar HRs were found in the included studies, reporting that individuals with increased oral hygiene practices had a lower risk of all-cause death (HR 0.76; 0.58–0.99) [45], CVD mortality (HR 0.25; 0.07–0.89) [76], MI (HR: 0.76; 0.62–0.94), and cerebral infarction (HR: 0.81, 95% CI: 0.69–0.94) [35]. However, oral health education is rarely provided for chronic disease management, and easily accessible information is scarce [6,7,8].

## 5. Clinical Significance

This review has highlighted that simple oral hygiene practices are associated with better cardiometabolic health outcomes and can reduce systemic inflammation. Therefore, non-dental clinicians are encouraged to recommend daily interdental cleaning followed by ≥2× daily TB to for at least two minutes for patients living with cardiometabolic conditions.

## 6. Strengths and Limitations

The strength of this systematic review is that it is the first review to summarise the impact oral health practices have on the risk, incidence, and mortality rate of CVD, DM, and/or CKD. This review followed a PROSPERO registered protocol. Two thirds of the included studies were rated as good quality. The global origin of the literature enables this review to be more generalisable. Although there was heterogeneity when reporting TB frequency, the overwhelming consensus found increased TB frequency (≥2× daily) and daily interdental cleaning had a positive effect on cardiometabolic disease outcomes.

However, there are limitations which need to be considered when interpreting the results. Many of the studies that reported on DM did not specify the type; it is therefore possible that the impact of oral hygiene varies depending on the condition of DM. Although the literature is global in nature, this review does not account for individual countries’ healthcare and belief systems, which may influence oral hygiene practices. Reporting bias is a part of all the included studies, as they relied on self-report data for oral hygiene practices. Furthermore, due to the heterogeneity within the study data collection and reporting, there is a need for further research using standardised oral hygiene questionnaires. Finally, and importantly, only three RCTs were identified. This highlights a great need for further clinical research to better understand causal relationships between oral health and cardiometabolic disease.

## 7. Conclusions

This systematic review has highlighted that for individuals living with or at risk of CVD, T2DM, and/or CKD, increased daily TB frequency (≥2× daily) as well as daily interdental cleaning is associated with reduced systemic inflammation, better health outcomes, and a lowered mortality risk. Cardiometabolic diseases are the leading causes of death and disability worldwide, and as such, provision of basic oral hygiene advice should be included in chronic disease prevention programs. Furthermore, development and implementation of interventional research involving cardiometabolic diseases and oral hygiene practices are needed to further strengthen the findings of this review.

## Figures and Tables

**Table 1:** Study characteristics.

First Author, Year, and Region	Focused Disease	Aim	Design	Sex (M%)	Population; Mean Age (Years)	Comorbidity CVD (Yes%)	Comorbidity DM (Yes%)	Comorbidity CKD (Yes%)	Oral Hygiene Practice(s)
Afsar 2013 [80]; Turkey	CKD	To determine regular TB frequency in HD patients and second, to determine factors related to regular toothbrushing in HD patients.	Observational	54%	135; 52.4 ± 13.6	32%	33%	N/A	TB frequency
Aggarwal 2012 [34]; India	T2DM	To investigate oral health attitudes, knowledge, and behavior with regard to diabetes-related factors among adults with T2DM and their attitudes to sustaining good oral health through proper oral hygiene and regular dental check-ups.	Cohort	53.2%	500; not reported	Not reported	N/A	Not reported	TB frequency Fluoridated Toothpaste use Mouthwash use Interdental cleaning
Almas 2003 [35]; Saudi Arabia	T2DM	To assess the effect of oral hygiene instructions on periodontal disease and to assess the glycemic changes in healthy, T2DM male Saudi patients.	Cohort	100%	60; 42 ± 13.6	Not reported	N/A	Not reported	TB frequency
Assante 2020 [33]; Brazil	ESKD	To correlate the self-reported oral health, oral hygiene, and dental assistance to clinic intercurrences with hospitalization in adult CKD patients under dialysis.	Cohort	49%	77; 54 ± 16.1	66.2%	2.6%	N/A	TB frequency Mouthwash use Interdental cleaning
Chang 2020 [37]; South Korea	CVD	To investigate the association of oral hygiene indicators with atrial fibrillation and heart failure risk in a nationwide general population-based longitudinal study.	Cohort	61.2%	161,286; 52.2 ± 8.7	HTN 38.9% DL 15.9%	9%	7.8%	TB frequency
Chang 2020 [32]; South Korea	DM	To assess the association between oral hygiene indicators and the risk of new-onset diabetes.	Cohort	57.9%	188013; 53.3 ± 8.6	HTN 34.6% DL 16.9%	0	0.2%	TB frequency
Chang 2021 [36]; South Korea	CVD	To investigate the relationship between the presence of periodontal disease and indicators regarding oral hygiene with occurrence for stroke in a nationwide population longitudinal cohort.	Cohort	59.4%	206,602; 53.5 ± 8.6	Hypertension 50.1% DL 24.5%	12.6%	0.3%	TB frequency
Chang 2021 [58]; South Korea	CKD	To clarify the relationship between oral hygiene indicators and CKD in a nationwide general population-based cohort.	Cohort	60.9%	158495; 52.3 ± 8.8	AF 0.4% HF 1.2% HTN 39% DL 24.5	8.8%	N/A	TB frequency
Cho 2021 [62]; South Korea	CVD	To investigate the association between periodontal health and stroke amount Korean adults.	Cross- sectional	47.3%	9497; 55.71 ± 0.17	Stroke 2.6% HTN 23.9%	9.4%	Not reported	TB frequency Oral hygiene product use
Choi 2019 [63]; South Korea	T2DM	To investigate the relationship between HbA1c level and self-reported periodontal symptoms among patients with T2DM.	Cross- sectional	22.4%	156; not reported	Not reported	N/A	Not reported	TB frequency TB technique
Cinar 2013 [79]; Turkey	T2DM	To assess the links between tooth loss, oral health behavior, T2DM, obesity, and sleep apnea among patients with T2DM.	Prospective intervention study	38%	165; not reported	Not reported	N/A	Not reported	TB frequency
Cinar 2014 [83]; Turkey	T2DM	Assess the impact of health coaching on TB self-efficacy and frequency, and effect on diabetics’ management and quality of life in comparison to health education among patients with T2DM.	RCT	Not reported	178; not reported	Not reported	N/A	Not reported	TB frequency
Cinar 2015 [64]; Turkey	T2DM	To assess the correlation between a preventive oral health behavior (toothbrushing), HDL, and self-assessed quality of life-related risk factors for non-communicable diseases and communicable diseases among patients with T2DM.	Cross-sectional	Not reported	178; not reported	Not reported	N/A	Not reported	TB frequency
deOliveira 2010 [65]; Scotland	CVD	To investigate whether self-reported frequency of TB (as a proxy of periodontal disease) was associated with risk of CVD events in a sample of adults from the Scottish Health Survey. To also examine the association between frequency of TB and inflammatory markers in a subset of participants.	Cross-sectional	46%	11869; 50.0 ± 11.0	HTN: 24%	2.6%	Not reported	TB frequency
deSouza 2014 [38]; Brazil	CKD	To assess the prevalence and impact of oral health parameters, periodontitis, and its treatment on survival in a group of patients undergoing hemodialysis.	Cohort	64.8%	122; 50 ± 13	23% HTN 75%	21%	N/A	Dental floss use
Frisbee 2010 [66]; United States	CVD	To investigate the associations between self-reported dental hygiene practices and overall dental health, cardiovascular risk factors and systemic inflammation, in adults living in rural communities.	Cross-sectional	37.5%	128; 41.5 ± 9.3	Not reported	Not reported	Not reported	TB frequency Flossing frequency
Fujita 2009 [67]; Japan	CVD, DM	To investigate the relationship between frequency of daily teeth brushing and prevalence of cardiovascular risk factors.	Cross-sectional	37%	54,551; not reported	HTN 41.5%	7.8%	Not reported	TB frequency
Guo 2023 [53]; China	CVD, DM	To evaluate the association between oral health behavior and various chronic diseases among middle-aged and older Chinese adults.	Cohort	48%	18,158; 61.3 ± 10.3	50.24%	40.2% grouped as diseases of the endocrine or nutritional metabolic.		TB frequency
Han 2021 [72]; South Korea	CVD, DM	To elucidate the prevalence of oral health complications and the relationship between DM and oral health status in diabetic patients with CVD.	Cross-sectional	42.9%	3495; not reported	Stroke 6.6% MI 3% HTN 71.2% DL 56.9%	24.2%	0.05%	TB frequency
Hiramatsu 2022 [81]; Japan	CKD	To investigate whether dental care in peritoneal dialysis patients might affect the incidence of CVD and infections such as peritonitis.	Retrospective observational	57.6%	165; Group A = 63.9 ± 12.0 Group B = 64.2 ± 10.5	Not reported	20%	N/A	TB frequency Dental floss frequency Mouth wash frequency
Hirano 2022 [39]; Japan	CKD	To evaluate the relationship between toothbrushing frequency and kidney function.	Cohort	50%	76,472; 45.9 ± 12.4	HTN 7.6%	2.24%	N/A	TB frequency
Huang 2023 [71]; Taiwan	CVD	To explore farmers’ self-care behaviors, including oral hygiene, remaining natural teeth, cardiometabolic risks, hepatitis, risk of stroke, and their determinant factors	Cross-sectional	54.8	183; 66.9 ± 11.7	Not reported	Not reported	Not reported	Dental floss use
Huh 2023 [57]; South Korea	CVD, T2DM	To evaluate the association of dental diseases and oral hygiene care (alone or in combination) with incident HF among patients with T2DM.	Cohort	72.4%	No PD: 62.8 ± 11.1 Yes PD: 65.3 ± 10.1	5.4%	N/A	0.84%	TB frequency
Hwang 2018 [68]; South Korea	CVD	To examine whether periodontal disease and/or poor oral health behavior predicted 10-year general cardiovascular risk among Korean adults with no CVD history using representative national data.	Cross-sectional	41.2%	8370; normal: 46.37 ± 11.92 At risk of CVD: 67.71 ± 10.6	HTN 22.4% DL 12.6%	8.29%	Not reported	TB frequency
Hwang 2022 [56]; South Korea	CVD	To investigate the association of periodontitis, missing teeth, and oral hygiene behaviors with the incidence of hypertension	Cohort	55.1%	104,349; 51.1 ± 8.2	Not reported	4.3%	Not reported	TB frequency
Isomura 2023 [54]; Japan	CVD	To investigate whether the timing of TB affects the risk of cardiovascular diseases.	Cohort study	58.2%	1583; reported as group medians Group MN: 65 Group night: 66 Group M: 68 Group none: 63	N/A	Not reported	Not reported	TB frequency
Jain 2015 [77]; India	CVD	To find the prevalence of periodontal disease in stroke patients and compare with age- and gender-matched controls and to compare the oral hygiene practices followed by stroke patients and controls.	Case–control	59.2%	216; 59 ± 12 (Stroke) 58 ± 9 (Control)	Stroke group only: AF 3.7% Hypercholesterolemia 9.25% HTN 72% Carotid stenosis 1.85% CAD 17.6% RHD 2.77%	44.4% (stroke group only)	0	TB frequency, device used, technique, timing, toothpaste use
Jangam 2017 [51]; United States	CVD	To examine the association between oral hygiene home care habits and cardiovascular mortality.	Cohort (part of a Ph.D. Thesis)	63.6%	506; 60	50.6% Hypertension 33.4%	9.8%	Not reported	TB frequency, instrument used, dental floss use, mouthwash use
Janket 2023 [76]; Finland	CVD	Primary: To determine if brushing and flossing affect the risk of CVD mortality in multivariable adjusted models. Secondary: To determine if mouthwash usage has independent impact on CVD mortality. To determine if mouthwash usage affects some periodontal pathogens and cariogenic bacteria proportions.	Case–control	68%	359; Seldom/no brushing: 56.3 ± 8.6 Daily brushing only: 58.0 ± 9.6 Daily brushing and flossing: 58.9 ± 8.0	CAD 44% HTN 31%	7.34%	Yes, number not reported	TB frequency Dental floss use Mouthwash use
Joshipura 2017 [41]; Puerto Rico	DM	To evaluate longitudinally the hypothesis that regular over-the-counter mouthwash use was associated with increased risk of pre-diabetes/diabetes over a three-year period.	Cohort	25.8%	945; 50.6 ± 6.8	Pre-hypertensive 30.7% HTN 47.2%	Prediabetic: 56.7%	0	Mouthwash use
Joshipura 2020 [42]; Puerto Rico	CVD	To evaluate if routine over-the-counter mouthwash use increases hypertension risk.	Cohort	22%	540; 49.0 ± 6.5	0	6.1%	0	Mouthwash use
Kaur 2023 [84]; India	T2DM	To evaluate the efficacy of subgingival home irrigation using water along with powered toothbrushes in diabetic patients with chronic periodontitis.	RCT	Not reported	31; Group A 50.13 ± 6.6 Group B 55.07 ± 9.83	0	N/A	0	TB instrument used Mouthwash use (Subgingival irrigation)
Kim 2022 [52]; South Korea	CVD	To investigate whether oral hygiene indicators were linked to the development of major CVDs in hypertensive patients in a longitudinal study setting.	Cohort	63.25%	52,677; 54.99 ± 9.51	AF 0.90%	7.21%	2.88%	TB frequency
Kobayashi 2020 [40]; Japan	CVD	Evaluate the association between the frequency of daily toothbrushing and subsequent cardiovascular events.	Cohort	50.3%	71,221; 45.6 ± 12.2	HTN 7.4% DL 4%	2.1%	Not reported	TB frequency
Kuwabara 2016 [69]; Japan	CVD, DM, CKD	To clarify the association between TB and risk factors for CVD: HT, DM, DL, HUA, and CKD	Cross-sectional	49%	85,866; 47 ± 11.5	HTN 15.9% DL 36.3%	4.3%	CKD 3.4% HUA 13.5%	TB frequency
Kuwabara 2017 [43]; Japan	CVD, DM	To clarify the relationship between TB practices and the risk factors for CVD: DM, DL, HT, and HUA.	Cohort	48.48%	13,070; not reported	HTN 19.9% DL 39.2%	4.4%	HUA 14.6%	TB frequency
Long 2023 [59]; China	CVD	To explore the relationship between oral health behavior and the incidence of stroke in Guizhou Province, China.	Cohort	47.5%	7970; 44.50 ± 15.15	HTN 26%	17.8%	Not reported	TB frequency
Luo 2022 [30]; United States	DM	To assess the association between inflammation and oral health and diabetes, as well as the mediating role of oral hygiene practice in this association.	Cohort	Not reported	2192; 64.5 (Elevated CRP) 62.4 (Non-elevated CRP)	0	No exact numbers reported	0	Dental floss use
Moon 2024 [73]; Korea	CVD	To explore the association between cardiovascular risk and frequency of toothbrushing in the context of traditional risk factors and inflammatory markers.	Cross-sectional	48.3%	13761; 51.3 ± 0.2	HTN 30.6%	11%	Not reported	TB Frequency
Matsui 2022 [55]; Japan	CVD	To determine the association of toothbrushing behavior assessed in detail with the incidence of future cardiovascular events in a general population including patients with CVD.	Prospective observational	68.9%	692; 63 ± 16	HTN 72% DL 60% CAD 19% HF 6.4% Stroke 8.4% Prior CABG 11%	27%	16% reported as dialysis	TB frequency and duration
Palmer 2015 [45]; Europe arm: (France, Hungary, Italy, Poland, Portugal, and Spain); and South American arm: (Argentina)	CKD	To investigate whether oral disease using standardized assessments of dental disease and preventative dental health practices are associated with early death in patients undergoing haemodialysis.	Cohort	57.7%	4205; 61.6 ± 15.6	MI: 12.5% Stroke: 10.3%	32%	N/A	TB frequency and duration Dental floss use Mouthwash use
Park 2019 [44]; South Korea	CVD	To evaluate whether oral hygiene behavior can alleviate cardiovascular risk associated with oral health status using a nationwide population-based cohort.	Cohort	58%	247,969; 52	HTN 19.9% DL 24%	6%	0.7%	TB frequency
Patel 2021 [78]; India	CVD	To assess and compare the periodontal health status among CHD patients with age- and gender-matched controls.	Case–control	63.6%	1616; 48.30 ± 7.73	50%	0	Not reported	TB frequency and method
Reichert 2015 [46]; Germany	CVD	To investigate whether oral hygiene habits, severe periodontitis, presence of periodontopathogens in the subgingival biofilm, or certain IL-6 genotypes represent independent risk factors for the incidence of new cardiovascular events among inpatients suffering from CHD.	Cohort	74%	942; 68.8 (median)	HTN 87.6%	34.20%	Not reported	Dental floss or interdental brush use TB frequency
Saengtipbovorn 2014 [82]; Thailand	T2DM	To assess the effectiveness of a Lifestyle Change plus Dental Care program to improve glycemic and periodontal status in the elderly with T2DM.	Quasi-experimental RCT	34.35%	131; Intervention: 63.83 ± 4.51 Control: 64.06 ± 5.53	0	N/A	0	TB Interdental cleaning Mouth rinse use
Song 2021 [47]; South Korea	DM, CVD	Evaluate periodontitis and poor oral hygiene as independent risk factors for either cerebral or MI in the diabetes population.	Cohort	64.75%	17,009; 55.65 ± 9.66	0	N/A	0	TB frequency
Song 2021 [48]; South Korea	DM (risk factor)	To assess the association between oral hygiene indicators of periodontitis, tooth loss, and TB with longitudinal fasting glucose levels in non-diabetic subjects.	Cohort	58%	91,963; 56.16 ± 7.6	HTN 36.2%	Not reported	9.1%	TB frequency
VanWormer 2013 [74]; United States	DM	To examine the degree oral hygiene habits were associated with CVD and T2DM risk levels among American adults.	Cross-sectional	43%	1008; not reported	0	0	0	TB and flossing frequency
Vogtmann 2017 [61]; Korea	CVD	To test the association between oral health, oral care, and denture use with overall and cause-specific mortality.	Cohort	46.4%	50024; 51.9 ± 8.9	HTN 30.6%	11%	Not reported	TB frequency
Wang 2022 [31]; China	DM, CVD (risk factor)	To investigate the relationship between oral health and incident hypertension/T2DM.	Cohort	47.5%	8139; 44.52 ± 15.16	HTN 26.1% DL 57.7%	8.4%	Not discussed	TB frequency
Winning 2017 [50]; UK	T2DM	The aim of this study was to investigate periodontitis as a risk factor for incident T2DM in a group of men aged 58–72 years.	Cohort	100%	63.7 ± 3.0	ACVD 9.09% HTN 29.45%	0	Not reported	TB frequency
Yoshioka 2021 [70]; Japan	T2DM	To clarify the association between oral health behavior and diabetes-related clinical indicators among patients with T2DM.	Cross-sectional	74.9%	74; 63.7 ± 12.4	HTN medications 56.8%	N/A	Not reported	TB timing
Zhang 2023 [75]; United States	DM	To examine the associations between oral health behaviors, specifically flossing and preventive dental care, and periodontitis and glycemic control, among US dentate adults with diabetes.	Cohort	51.1%	892; 60.0 ± 0.4	Not reported	100%	Not reported	Flossing
Zhuang 2021 [49]; China	CVD, T2DM, CKD	To investigate the associations between a self-reported measure of TB behavior [as a proxy of oral hygiene] and the risk of cardiovascular events, as well as examine the association of TB behavior with nonvascular diseases such as DM and CKD.	Cohort	41%	487,198; 51.5 ± 10.6	0	3.2%	Not reported	TB frequency
Zhou 2024 [60]; China	CVD	To examine the associations of oral health with all-cause and cause-specific mortality in middle-aged and older Chinese adults.	Cohort	27.6%	28006; 62.0 ± 7.1	Not reported	14.6%	Not reported	TB frequency

%: percentage; ACVD: acute cardiovascular disease; CABG: coronary artery bypass graft; CAD: coronary artery disease; CHD; coronary heart disease; CKD: chronic kidney disease; CRP: C-reactive protein; CVD: cardiovascular disease; DL: dyslipidemia; DM: diabetes mellitus; DM: diabetes mellitus; ESKD: end-stage kidney disease; HD: haemodialysis; HDL: high-density lipoprotein; HTN: hypertension; IL-6; cytokine interleukin-6; MI: myocardial infarction; RCT: randomised controlled trial; RHD: rheumatic heart disease; T2DM: type II diabetes mellitus; TB: toothbrushing.

**Table 2:** Study findings.

First Author and Year	Strategy	Comparator/Variable	Findings
Afsar 2013 [80]	OR and 95% CI	Regular TB vs. Irregular TB	TB was infrequent in study participants. Depression, cognitive function, education, and chronic inflammation influenced TB in participants. Serum hs-CRP levels showed inverse association with regular TB.
Aggarwal 2012 [34]	% and *p* values.	Good DM Control HbA1c ≤ 6.0% Moderate DM Control HbA1c 6.1–7.5% Poor DM Control HbA1c ≥ 7.5%	Maintenance of good oral hygiene was poor among participants. 49% reported TB once daily. 2× daily TB was lowest within the poor HBa1c category. Patients without DM complications had a higher rate of 2× daily TB (*p* < 0.001). Only 38.4% patients were aware that diabetes affects oral health. 52.8% reported that their dentist did not know they had diabetes.
Almas 2003 [35]	Mean ± SD, %, and *p* values.	Group 1: Healthy [non-diabetic] with periodontal disease Group 2: T2DM with early to moderate periodontitis Group 3: T2DM with advanced periodontitis	HbA1c and GCF levels decreased significantly after one week of oral hygiene instructions: HbA1c G1: *p* < 0.004 G2: *p* < 0.002 G3: *p* < 0.000 GCF G1, G2, G3: *p* < 0.000 The severity of periodontal disease increased with an increase in HbA1c and GCF levels. * Limited change in CPITN measurements. * Mean plaque score decreases in all groups, with the largest in group 3. Structured oral hygiene instructions can have a positive effect on the metabolic control of type 2 diabetes. * Exact numbers not reported.
Assante 2020 [33]	Pearson’s Correlation [r]	TB times per day Use of Dental Floss Use of Mouthwash	Strong correlations between oral hygiene habits and intercurrent hospitalization of ESKD patients undergoing dialysis. Those not using dental floss and mouthwash needed 7.7% and 30.8% more hospitalizations than those who were using floss and mouthwash, respectively. Negative correlation [r = −0.89] between times of TB per day and patient hospitalization. Negative correlation [r = −0.98] between oral health self-report and patient hospitalization. Positive correlation [r = 0.9] between number of remaining teeth and oral diseases.
Chang 2020 [37]	HR Kaplan–Meier survival curves	TB times/day: 0–1× daily, 2× daily, ≥3× daily.	The risk of AF and HF was lower in groups with frequent TB. Fewer teeth was associated with increased risk of AF and HF. Kaplan–Meier survival curves showed that the risk for AF and HF was lower in subjects without periodontal disease. Frequent TB (≥3× daily) was significantly associated with attenuated risk of AF and was related with decreased risk of HF occurrence. Optimal oral health, gained by frequent TB may decrease risk of AF and HF.
Chang 2020 [32]	HR	Periodontal disease No Yes Teeth brush times/day 0–1 2 ≥3 Dental visit history No yes Dental scaling No Yes Number of missing teeth 0 1–7 8–14 ≥15	16.1% of cohort developed new-onset DM at the 10-year follow-up. Frequent TB may reduce the risk of new-onset DM. The presence of periodontal disease and lower remaining teeth may increase the risk of new-onset DM. After accounting for all confounders: Frequent TB (≥3× daily) was negatively correlated with occurrence of new-onset DM (*p* < 0.001). Periodontal disease was associated with new-onset DM (*p* < 0.001). Number of missing teeth (≥15 teeth) was positively associated with occurrence of new-onset DM (*p* < 0.001).
Chang 2021 [36]	HR Kaplan–Meier survival curves	Periodontal disease No Yes Teeth brush times/day 0–1 2 ≥3 Dental visit history No yes Dental scaling No Yes Number of missing teeth 0 1–7 8–14 ≥15 Number of dental caries 0 1–3 ≥4	Frequent TB (≥3× daily) was related to decreased risk of stroke (*p* < 0.001) In the sensitivity analysis, frequent TB was associated with risk of cerebral infarction. Only TB frequency (≥3× daily) was associated with risk of cerebral and subarachnoid hemorrhage in the multivariable analysis. Kaplan–Meier survival curves demonstrated that the risk for stroke depended on number of daily TB, dental visit history, dental scaling, number of teeth loss, and number of dental caries. Regular and frequent daily oral hygiene care were negatively and infrequent oral hygiene care positively related with risk of occurrence for stroke, respectively.
Chang 2021 [58]	t-tests and logistic regression analysis were performed for statistical analysis	Periodontal disease No Yes Teeth brush times/day 0–1 2 ≥3 Tooth loss 0 1–7 8–14 15–21 ≥22 Professional scaling No Yes	Increased TB frequency may reduce the risk of CKD. Kaplan–Meir survival curves showed periodontal disease and fewer remaining teeth were positively related with an elevated risk of CKD (*p* =< 0.001). The risk of CKD had a negative correlation as the frequency of TB (*p* =< 0.001). Increased number of TBs (≥3 times a day) was inversely associated with the development of CKD. The subgroup analysis found having BMI ≥ 25 and TB at least 3 times a day were more strongly associated with occurrence of CKD (*p* for interaction < 0.05).
Cho 2021 [62]	OR Logistic regression analysis	Daily TB 0 1–2 ≥3 Use of oral hygiene products 0 1–2 ≥3 Self-perceived oral health Poor Good Tooth ache Yes No Chewing level Poor Fair Good Untreated tooth Poor Good Number of residual teeth 0–19 ≥20 CPI 4 3 2 1 0	Daily TB frequency had a significant negative effect impact on periodontal health and stroke. The stroke group showed lower daily TB frequency and less use of other oral hygiene products than the no-stroke group (*p* < 0.001).
Choi 2019 [63]	OR	TB/day ≤2 ≥3 TB technique Non-rolling stroke rolling stroke Recent dental checkups <2 years ago >2 years ago Interproximal cleaning devices No Yes Periodic dental scaling No Yes HbA1c level Good control [<7.0] Poor control [≥7.0]	HbA1c control and experiencing periodontal symptoms were correlated in patients ≥ 50 with T2DM. Participants who brushed their teeth ≥ 3 times daily had more periodontal symptoms (*p* < 0.05). Participants with poorly controlled HbA1c level had more periodontal symptoms than those with good control (*p* < 0.05). * * Authors do not disclose/define periodontal symptoms. Does not correlate with TB frequency and T2DM.
Cinar 2013 [79]	Pearson’s Correlation (r)	No. teeth lost in upper jaw <mean ≥mean No. teeth lost in lower jaw <mean ≥mean Total no. teeth lost <mean ≥mean TB frequency Daily Less than daily Gingival bleeding on TB Rare At least occasionally	Oral care with early diagnosis and monitoring of glycemic level can help prevent complications of T2DM. TB frequency was negatively correlated with fasting blood glucose (r_s_ = 0.18, *p* < 0.005) and positively with HDL (r_s_ = 0.23, *p* < 0.001]. Patients who TB less than daily have an increased risk of HDL (≤39 mg/dL, %), 78%, compared to those who TB daily, 53% (*p* < 0.05). Patients who TB less than daily have an increased risk of LDL (≥95 mg/dL, %), 50%, compared to those who TB daily, 45%. Patients who TB less than daily have an increased risk of HbA1c (≥6.5%), 79%, compared to those who TB daily, 68%. Patients who TB less than daily have an increased risk of probability of sleep apnea, 47%, compared to those who TB daily, 30% (*p* < 0.05).
Cinar 2014 [83]	Compared numerical data, percentage, and used *p* values.	Health Coaching Health Education	70% of HC participants increased their TB 2× daily (*p* < 0.05). 0.6% improvement in HbA1c levels observed in the HC group (*p* < 0.05). HC is more effective at improving TB behavior than HE, contributing significantly to DM management.
Cinar 2015 [64]	OR Pearson’s Correlation (r)	TB frequency Daily Less than daily Gingival bleeding on TB Favorable Unfavorable	27% of participants brushed 2× daily. Self-reported gingival bleeding was negatively correlated with QoL and TB frequency. TB was positively correlated with HDL and QoL. Participants who brushed < 1× daily were more likely to have unfavorable HDL and QoL than those who brushed daily [OR = 1.84 CI = 1.02–3.32 vs. OR 1.53 CI = 1.07–2.18, *p* < 0.05].
deOliveira 2010 [65]	HR Regression models	TB frequency 2× daily 1× daily <1× daily	TB is associated with CVD. Participants who reported less frequent TB had a 70% increased risk of a cardiovascular disease event. Less frequent TB was associated with increased concentrations of CRP and fibrinogen.
deSouza 2014 [38]	Uni- and multivariate analysis HR Kaplan–Meier Curves	Frequency of dental visits Rarely Annually/biannually Quarterly Use of dental floss Decayed teeth Filled teeth	Oral health status was poor amongst participants. Dental floss use was irregular in 40% of participants. The univariate analysis showed a lack of dental floss use and low frequency of visits to the dentist were positively associated with an increased risk for patient mortality (HR 1.99 [0.85–4.14] *p* = 0.066). After the multivariate analysis, the significance of this correlation disappeared (HR 0.19 [0.2–3.10] *p* = 0.731).
Frisbee 2010 [66]	Univariate analysis using Pearson’s chi-square statistic. Univariate ANOVA analysis	Frequency of TB At least daily ≤1× daily Frequency of flossing 2–6 times a week ≤1× a week Frequency of dental care Every 6 months Less than 6 months	Dental hygiene (defined as TB, flossing preventative care) was significantly related to total cholesterol, mean arterial pressure, adiponectin, CRP, fibrinogen, IL-1B, and tPAI-1. In both analyses, there was a statistically significant independent association between dental hygiene and systemic inflammation. Systemic inflammation from periodontal disease could begin before the onset of the clinical signs associated with poor dental hygiene. Better overall dental health was associated with lower levels of CRP levels and higher levels of adiponectin and fibrinogen.
Fujita 2009 [67]	Kappa coefficient was calculated. Pearson’s chi-square tests OR	Frequency of TB After every meal 1–2 times a day Hardly ever	Lower frequency of daily TB was significantly related to higher prevalence of DM and high TG and/or low HDL-C in both men and women, as well as a higher prevalence in hypertension for women. Total cholesterol status was not significantly affected by TB frequency.
Guo 2023 [53]	Multivariate logistic models Logistic regression OR	Frequency of TB ≥2× daily ≤1× daily	Poor oral hygiene practices were associated with higher risk of chronic diseases. TB ≤1× daily increased the risk of hypertension, ischemic heart disease, cerebrovascular disease, and diabetes.
Han 2021 [72]	Kolmogorov−Smirnov test Chi-square test, Independent t-test Logistic regression	No DM DM	The DM group had a significantly higher number of people with a lower TB frequency (*p* < 0.001) compared with the non-DM group. There was a correlation between increased prevalence of oral health diseases and fewer remaining teeth in those living with the combination of CVD and DM. The incidence of periodontitis was found to be 1.4 times higher in the DM group compared to the non-DM group after adjusting for confounding variables.
Hiramatsu 2022 [81]	Compared numerical data, percentage, and used *p* values. ANOVA testing Regression analysis	Dental care score ≥ 7 Dental care score < 7	Oral hygiene practices were grouped and given a dental score. These scores then stratified the groups by Group A (≥7-good dental care) and Group B (<7 poor dental care). Participants in Group B had lower daily TB frequency than those in Group A [1.33 ± 0.58 vs. 2.54 ± 0.50 *p* = 0.001]. Increased albumin and lower CRP were associated with Group A. The multiple regression analysis found TB frequency was the most important factor for all-cause peritonitis [t value = −2.182, *p* = 0.048], streptococcal peritonitis [t value = −2.739, *p* = 0.006], CHF [t value = −1.739, *p* = 0.059], and pneumonia [t value = −2.916, *p* = 0.004].
Hirano 2022 [39]	Multivariate analysis Compared numerical data and percentage and used *p* values.	TB frequency Once a day or not everyday 1–2 times/day After every meal	Participants who brushed 1–2× daily had a lower prevalence of cancer and DM. Participants who brushed after every meal had a lower prevalence of hypertension. Those who brushed their teeth 1–2× daily had a higher eGFR and milder stage of CKD than those who brushed their teeth ≤ 1× daily. Those who brushed their teeth after every meal fell within a more severe CKD risk category, suggesting more severe proteinuria, than those who brushed their teeth 1–2× daily. Frequent TB was associated with a decreased risk of composite renal outcomes defined by a 25% eGFR reduction from baseline values, an eGFR of <15 mL/min/1.73 m^2^ (stage 5 CKD), and a requirement for regular dialysis. TB 1–2× daily was associated with better kidney function than TB after every meal.
Huang 2023 [71]	Compared numerical data and percentage and used *p* values. Hierarchical multiple linear regression analyses	Dental floss use before bed Seldom/occasional Usually/always	Having a lower remaining teeth was significantly associated with seldom or occasional dental floss use after dinner/before bed [t = 4.93, *p* < 0.001], and high risk of stroke [t = 8.38, *p* < 0.001]. The regression analyses showed that the determinant factors associated with the remaining natural teeth were high risk of stroke [t = −2.6, *p* < 001], and dental floss use [t = 1.98, *p* < 0.05].
Huh 2023 [57]	Kaplan–Meier curves HR Compared numerical data	Periodontal disease No Yes Dental caries No Yes No. missing teeth 0 1–7 8–14 ≥15 Professional dental cleaning <1 yearly ≥1 yearly Daily TB 0–1 ≥2	Oral diseases and lower TB frequency were associated with HF incidence amongst patients with T2DM. HR for HF decreased by 10% among participants who brushed their teeth ≥ 2 daily (HR 0.90 [95% CI, 0.82–0.98] *p* = 0.018). Individuals with ≥15 missing teeth and lower TB frequency had an increased HF risk (HR 1.50 [95% CI, 1.09–2.06] for 0–1 time of daily TB). These associations were not observed among individuals with ≥15 missing teeth and higher TB frequency (HR 1.16 [95% CI, 0.92–1.47] for ≥2 times of daily TB). The HRs for HF increased by 20% among individuals with both periodontal diseases and dental caries (HR 1.20 [95% CI, 1.01–1.43]; 5-year RD 0.20, SE 0.10).
Hwang 2018 [68]	OR Compared numerical data and percentage and used *p* values.	Perceived oral health. Good Moderate Not good Diagnosed periodontal disease Yes No Frequency of TB ≥3 times a day <3 times a day Dental visit experience in a year Yes No Using oral hygiene supplies Yes No Preventative dental treatment Yes No	TB < 3 times per day was an independent predictor of intermediate/high CVD risk. Diagnosed periodontal disease and low TB frequency predicted a higher 10-year cardiovascular risk among people with no history of CVD.
Hwang 2022 [56]	HR Log-rank test Compared numerical data and percentage and used *p* values.	Periodontal disease No Yes Number of missing teeth 0 1–7 8–14 ≥15 Dental scaling No Yes Teeth brush times/day 0–1 2 ≥3	TB frequency (≥3 daily) at baseline (HR: 0.85) was significantly associated with incident hypertension. Periodontitis was significantly associated with incident hypertension (HR: 1.02; 95% CI: 1.00–1.04). The risk of hypertension was lower in the higher frequency TB group (≥3 vs. 0–1 times/day; HR: 0.85; 95% CI: 0.83–0.88; *p* for trend < 0.0001). In the middle-aged group (40–64), frequency of TB [for ≥3 group; HR: 0.85; 95% CI: 0.83–0.88] was significant. In the older group (≥65 years), weaker effects were observed for frequency of TB (for ≥3 group; HR: 0.93; 95% CI: 0.87–0.99). Protective effects of TB disappeared in the group with fewer remaining teeth and periodontitis.
Isomura 2023 [54]	Kaplan–Meier curves HR Compared numerical data	TB frequency: Group MN (morning and night) Group M (morning only) Group Night (night only) Group None (not brushing)	Univariate and multivariate analyses of cardiovascular events showed significantly higher survival estimates in Groups MN (*p* = 0.021) and Night (*p* = 0.004) than in Group None. TB only in the morning is inadequate to prevent CVD. There were no significant differences in cardiovascular events, hospitalization events, or life prognosis among the groups based on the number of teeth, caries, or periodontal disease.
Jain 2015 [77]	Chi-square and Fisher’s exact tests OR	Type of teeth cleaning Toothbrush Finger/twig Method of cleaning Horizontal Vertical/circular Material used Toothpaste Other Frequency of cleaning/day Once More than once Time of TB Before meals After meals Decayed teeth Missing teeth Filled teeth	Those living with stroke were more likely to use finger/twig to clean their teeth compared to the control group (17.4% vs. 1.9% [*p ≤* 0.0001]). Those living with stroke were less likely to brush more than once a day compared to the control group (7.7% vs. 26.9% [*p* < 0.0001]). Those living with stroke had a greater number of missing teeth compared to control group [(4.02 ± 5.85) vs. (1.81 ± 3.89) (*p* = 0.002)]. Those with poor oral hygiene practices were significantly more likely to develop stroke and periodontal disease.
Jangam 2017 [51]	Univariate analysis T-test/Chi-squared test Multiple regression model Logistic regression model OR *p* value	Oral Hygiene Score 0 = low 1 = medium 2 = high Dental check-ups 1 = once a year >1 = longer duration between check-ups Electronic toothbrush and sweets Yes No Sugary beverage intake Yes No Number of teeth	An inverse correlation exists between oral hygiene habits and CVD mortality (*p* = 0.02). All oral hygiene habits, particularly flossing (*p* = 0.03), decreased the risk of CVD outcomes. * There was an association between individual oral habits and CVD: flossing, TB, number of teeth, last dental visit, and use of electronic toothbrush were each associated with CVD. However, mouthwash use (*p* = 0.60) was the only oral habit that had no independent association with CVD. This study found that the odds of a person with a high OH score (OR = 0.21) dying due to CVD were lower compared to those with a low oral hygiene score (OR = 0.59). * CVD outcomes not defined.
Janket 2023 [76]	HR Compared numerical data Kaplan–Meier curves	Oral hygiene self-care practice: Better (daily TB and flossing) Good (daily TB only) Poor (no TB/flossing) Baseline CAD Mouthwash use No Yes	Daily TB and flossing had significantly greater benefits on CVD mortality risk, over TB alone. CVD mortality risk was the lowest in the best OHS group (both TB and flossing) (HR = 0.25 [CI: 0.07–0.89]; *p* = 0.03) and in the TB-only group (HR = 0.72 [CI: 0.37–1.41]; *p* = 0.34). In a stratified analysis, the CAD group had enough CVD mortality and beneficial effects of OHS remained (HR = 0.50 [0.24–1.06]; *p* = 0.07). CRP was marginally significant between groups. SLZ (an innate oral immunity marker) had a significant inverse correlation with OHS. The effect of independent mouthwash usage on CVD mortality was not meaningful (HR 0.95 [0.45–2.01]; *p* = 0.89).
Joshipura 2017 [41]	Poisson regression models IRR Percentage comparison Confidence intervals	Mouthwash use frequency Never <1× weekly 1 time per week 2 times per week 1–3× weekly 4–6× weekly 1× daily 2× daily ≥2× daily	Controlling for major DM risk factors, participants who used mouthwash ≥ 2× daily had a 55% increased risk of developing pre-DM or DM over a 3-year follow-up compared to less frequent users, and 49% higher risk compared to non-users of mouthwash. The authors hypothesize that the increased risk for diabetes among mouthwash users in their study is due to direct effects on the oral microbiome.
Joshipura 2020 [42]	Poisson regression models IRR Linear regression Percentage comparison Confidence intervals	Mouthwash use frequency ≤6× weekly 1× daily ≥2× daily	People who used mouthwash ≥2× daily had an 85% higher risk of being diagnosed with hypertension compared to less frequent users, and more than twice that of non-users. In unadjusted models, ≥2× daily use of mouthwash was associated with an increased risk of being diagnosed with hypertension (IRR = 1.87, 95% CI: 1.07, 3.27), compared to no use. Only using mouthwash 1× daily was associated with increase in SBP over follow-up (β = 3.98, 95% CI: 0.83, 7.13) compared to no use. Compared to no mouthwash use, its use once daily (β = 2.31, 95% CI:0.24, 4.38) and twice daily (β = 2.29, 95% CI: 0.30, 4.27) were associated with increased DBP. Almost all types of mouthwash are likely to have a detrimental impact on nitrate reducing bacteria, which may increase BP.
Kaur 2023 [84]	Mean ± SD values Independent samples *t*-test Pairwise inter group comparison Mann–Whitney test Spearman correlation	Mouthwash: Group A:Waterpik + 0.06% CHX Group B: CHX rinse 0.12% TB: Group A: Powered toothbrush Group B: Manual	Significant reduction in HbA1c levels at final follow-up (4 months) in both groups compared to baseline; Group A *p* = 0.003, Group B *p* = 0.008. Non-significant reduction in CRP and IL-B at final follow-up from baseline.
Kim 2022 [52]	HR Cox proportional hazard regression Compared numerical data and percentage and used *p* values. Cumulative incidence curves	Periodontitis No Yes Number of missing teeth 0 1–4 ≥5 Number of dental caries 0 1–4 ≥5 Teeth brush times/day 0–1 ≥2	Frequency of TB per day (at least twice) was significantly related to a lower risk of major cardiovascular events (aHR: 0.88; 95% CI: 0.81–0.96; *p* = 0.002). In the secondary analysis for individual cardiovascular outcomes, TB frequently (at least two times per day) was associated with a lower risk of all stroke (aHR: 0.87; 95% CI: 0.79–0.96; *p* = 0.004) and hemorrhagic stroke (aHR: 0.77; 95% CI: 0.64–0.92; *p* = 0.003) compared with TB teeth 0–1 times per day.
Kobayashi 2020 [40]	Bivariable analyses Chi-square tests OR	Frequency of TB Not every day Once a day Once to twice a day After every meal	Frequent TB was inversely associated with subsequent cardiovascular events in a dose-dependent manner. Participants who brushed 1× daily may have an increased risk only for stroke, but not for ACS, compared to those who brushed their teeth after every meal. Less frequent TB may be a marker for subsequent CVD and CHD, rather than a risk factor.
Kuwabara 2016 [69]	ANOVA Binominal logistic regression models OR *p* value and confidence intervals	TB practices After every meal At least 1× daily <1× daily	Adjusted analyses found low-frequency TB was significantly associated with high prevalence of T2DM (OR = 2.03) and dyslipidemia (OR = 1.50) but not with hyperuricemia and CKD. Higher TB frequency may be beneficial for improving oral health and preventing some systemic diseases.
Kuwabara 2017 [43]	Logistic regression analysis OR Percentage comparison *p* value	TB practices After every meal At least 1× daily <1× daily	Low-frequency TB was an independent risk factor for developing DM in males (OR: 1.43) and for developing DL in females (OR: 1.18). Low-frequency TB was not a risk factor for developing DM in females, DL in males, hypertension, and HUA. TB practices may be beneficial to reduce CVD risk factor development.
Long 2023 [59]	Cox regression, HR	TB Frequency <1× daily 1× daily ≥2× daily	Compared to <1× daily brushing, the risk of all-cause stroke decreased by 34% (HR = 0.65, 95% CI: 0.45–0.94) for those TB 1× daily and by 50% (HR = 0.49, 95% CI: 0.30–0.78) for those TB ≥ 2× daily. Compared to <1× daily TB, the risk of ischemic stroke decreased by 29% (HR = 0.71, 95% CI: 0.46–1.08) for those TB 1× daily and by 49% (HR = 0.51, 95% CI: 0.29–0.89) for those TB ≥ 2× daily. There were no significant associations found between oral hygiene behavior and hemorrhagic stroke.
Luo 2022 [30]	Chi-square tests and t-tests Linear regression model Logistic regression model OR *p* value	Periodontitis Yes = 1 (mild, moderate, or severe) No = 0 STL (<20 teeth out of 28 teeth) Yes No	Having both STL and DM (b = −2.64), having STL alone (b = −2.08), and having DM alone (b = −1.09) were negatively associated with flossing, suggesting that those with STL or DM were less likely to floss frequently. Significant tooth loss is associated with higher systemic inflammation. Flossing may contribute to reducing systemic inflammation.
Moon 2024 [73]	ANOVA or Kruskal Walls test Chi-square test Tukey HSD or Steel–Dwass test Cox proportional hazards regression analysis *p* value and confidence intervals HR	TB frequency and duration Low frequency and short duration Low frequency or short duration Non-low frequency and non-short duration	The combination of decreased frequency and duration of TB is associated with a higher risk of cardiovascular events. Participants in the low-frequency and short-duration group had significantly higher circulating hs-CRP levels compared to the non-low and non-short group (*p* = 0.04). There were no significant differences in the incidences of MACEs between the non-low-frequency and non-short-duration group and the low-frequency or short-duration group.
Matsui 2022 [55]	Mean ± standard errors, and percentages. Generalized linear models Multivariable adjustment models	TB Frequency 0–1 daily 2 daily ≥3 daily	Those who reported more frequent toothbrushing had the following: lower systolic BP; lower non-HDL cholesterol, triglyceride, and fasting plasma glucose levels; higher HDL cholesterol levels; however, LDL cholesterol levels did not differ significantly. The estimated 10-year ASCVD risk was 13.7%, 9.1%, and 7.3% for those who brushed their teeth 0–1, 2, and ≥3 times a day, respectively. Participants who reported brushing their teeth ≥ 3 times/day had 0.48 mg/L and 0.32 mg/L lower levels of hsCRP than those who brushed their teeth 2 times/day and 0–1 time/day, respectively (unadjusted *p* values < 0.001 for both). WBC levels showed significant differences according to toothbrushing behaviors after multivariable adjustment (−0.20 × 10^3^/µL, *p* = 0.013; and −0.18 × 10^3^/µL, *p* = 0.021, respectively).
Palmer 2015 [45]	*p* value Kaplan–Meier curves Multivariable-adjusted Cox proportional hazards regression models	DMFT Very low (<5) Low (5–8.9) Moderate (9–13.9) High (14 or more)	TB daily was associated with longer survival for all-cause death (HR 0.76 (0.58–0.99)); however, it was not associated with a lower risk of cardiovascular death (HR 0.74 (0.52–1.07)). Flossing was associated with longer survival aHRs of 0.52 (95%CI, 0.32–0.85) and lower risk of cardiovascular death 0.25 (0.09–0.68). Mouthwash use was associated with longer survival aHRs of 0.79 (0.64–0.97) and lower risk of cardiovascular death 0.60 (0.44–0.82). TB for ≥2 min was associated with longer survival aHR of 0.76 (0.58–0.99) and lower risk of cardiovascular death aHR 0.74 (0.52–1.07). Tooth loss and dental disease were associated with all cause and cardiovascular mortality, while better oral hygiene practices were associated with better survival in those living with ESKD being treated with HD.
Park 2019 [44]	Kaplan–Meier curves HR CI Multivariable regression models	Number of dental caries 0 1–5 ≥6 Number of lost teeth 0 1–7 8–14 15–21 22–28 Number daily TB 0–1 2 ≥3 Professional clean <1× a year ≥1 a year	TB one more time a day was associated with a 9% lower risk of cardiovascular events (HR: 0.91; 95% CIs: 0.89–0.93; *p*< 0.001). TB three times a day or more was associated with a 19% reduction in cardiovascular events compared with TB once or less a day. Better oral hygiene practices and regular preventative dental visits are associated with a lower cardiovascular risk. The benefit of oral hygiene is independent of oral hygiene status and other confounding factors.
Patel 2021 [78]	Chi-square test Mann–Whitney U test ANOVA Regression analysis OR	TB method Toothbrush and paste Other TB frequency Once daily ≥2× daily	TB frequency showed a significant association with CHD (*p* = 0.001). Using other TB aids (neem sticks, miswak, etc.) showed lower risk of developing CHD (OR = 0.71); however, upon adjusting, it showed a 1.15 times higher risk for CHD. Lower TB frequency was associated with 3.38 times higher risk for CHD (OR = 3.38; adjusted OR = 2.54).
Reichert 2015 [46]	Kolmogorov–Smirnov test Shapiro–Wilk test Mann–Whitney U HR Log-rank Kapal–Meier Curves	Use of dental floss/interdental brushes No Yes	Dental flossing and interdental brush use might reduce the risk of new CVD events amongst individuals living with CHD. For those that used dental floss or interdental brushes, the bivariate analyses found significantly lower incidence for endpoints MI, stroke/TIA and death from cardiovascular causes, compared to those who did not use them (1.6% vs. 8.8%, log-rank *p* = 0.001). The use of dental floss and/or interdental brushes was significantly associated with a decreased risk of new cardiovascular events among those living with CVD after 1 year (*p* = 0.01). Dental floss/interdental brush use had an inverse association with severe periodontitis prevalence. TB frequency was not found to impact CVD outcomes.
Saengtipbovorn 2014 [82]	Compared numerical data and percentage and used *p* values. Descriptive statistics, Chi-square test, Fisher’s exact test, Multiple linear regression	Intervention group (LCDC) Control group	After the LCDC program, FPG and HbA1c decreased with statistically significant differences between the intervention and the control groups at 3 month follow-up (*p* = 0.003 and *p* =< 0.001, respectively). After the 3-month follow-up, the participants in the intervention group were more likely to use mouth rinse, salt solution, dental floss, and an inter-proximal brush. However, only salt solution and dental floss had statistically significant differences (*p* = 0.020 and < 0.001, respectively). The combination of LCDC in one program, improved both glycemic and periodontal status in the elderly with T2DM.
Song 2021 [47]	Compared numerical data and percentage and used *p* values. Kaplan–Meier curves HR Multivariate analysis	Periodontitis No Yes Number of missing teeth 0 1–4 ≥5 Number of dental caries 0 1–4 ≥5 Teeth brush times/day 0–1 ≥2	Frequent TB was associated with a lower risk of cerebral or myocardial infarction in individuals living with DMs. TB 2× daily was significantly associated with a lower risk of both cerebral aHR: 0.81 (95% CI: 0.69–0.94); *p* = 0.007, and myocardial aHR: 0.76 (95% CI: 0.62–0.94); *p* = 0.012 infarction. Oral diseases are significantly associated with long-term cardiovascular events, particularly cerebral infarction.
Song 2021 [48]	Compared numerical data and percentage and used *p* values. Descriptive statistics, Chi-square test Linear mixed model	TB frequency 0–2× daily ≥3× daily	Compared with TB ≤ 2 times per day, TB ≥ 3 times per day was significantly associated with decreased fasting glucose levels (β = −0.0207 mmol/L, SE = 0.0033, *p* < 0.001). The authors suggest that poor oral hygiene is a potential therapeutic target for glycemic control, implying that interventions to improve oral hygiene, including frequent TB, may improve long-term fasting glucose regulation.
VanWormer 2013 [74]	Compared numerical data, regression modeling. Point estimate ± standard error, *p* value	Oral hygiene index classified as follows: Excellent, good, fair, or poor. Classifications were made depending on the following factors: TB frequency <1× daily 1× daily ≥2× daily Flossing Frequency Never Rarely Some days Most days Daily	The median CVD risk score was 6.2% (range 0.4–55.8%). The initial crude model indicated a significant association between oral hygiene (F = 3.60, *p* = 0.018) and CVD risk score. Sensitivity analysis in this study indicated that both toothbrushing and flossing were independently associated with CVD risk score, but these predictors did not interact. Participants brushing ≥ 2× daily had a lower risk (approx. 9%) of a CVD event at 10 years compared to those brushing < 1× daily (approx. 12%). Participants flossing daily had a lower risk (approx. 8%) of a CVD event at 10 years compared to those rarely flossing (approx. 13%). The median T2DM risk score was 9.8% (range 0.1–94.2%). The initial crude model did not indicate a significant association between oral hygiene and T2DM risk score (F = 1.65, *p* = 0.188). The authors suggest this is due to selection bias of participants, thus leaving a smaller sample size.
Vogtmann 2017 [61]	Compared numerical data, percentage, *p* values and HR used. Cox proportional hazards	TB frequency Never 1× daily ≥2× daily Other Quintile of tooth loss Q1 (Fewer teeth missing than expected) Q2 Q3 (expected number of teeth missing) Q4 Q5 (More teeth missing than expected)	TB was inversely associated only with CVD mortality. Although brushing once a day, aHR 0.79 [95% CI: 0.69, 0.91], and the “other” category of brushing, aHR 0.76 [95% CI: 0.65, 0.88], had an inverse association, brushing twice a day was not associated with cardiovascular disease mortality, aHR 1.06 [95% CI: 0.84,1.32], compared with never brushing. Any type of TB decreased the risk of overall mortality compared with never brushing. Participants in Q5 and/or who used dentures had a higher risk of overall mortality in both adjusted and unadjusted models.
Wang 2022 [31]	Compared numerical data and percentage and used *p* values. HR Multivariable regression models	TB frequency 0× daily <1× daily 1× daily ≥2× daily	Frequent TB was associated with a reduced risk of hypertension and DM incidence. Compared with those never brushing, participants TB ≥ 2× daily had an 0.54 (95% CI: 0.40–0.72; *p* < 0.001) times decreased risk of hypertension and adjusted 0.64 (95% CI: 0.44–0.93; *p* < 0.001) times decreased risk of DM. TB at least twice a day may prevent future hypertension and DM events.
Winning 2017 [50]	Compared numerical data and percentage and used *p* values. Kaplan–Meier curves HR	Periodontal status No/mild periodontitis. Moderate periodontitis Severe periodontitis	TB frequency as part of Model 1, 2, 3, and 4 in the dose–response risk for incident T2DM was significant when comparing severe with no/mild periodontitis. Baseline moderate to severe periodontitis was an independent risk predictor of incident T2DM.
Yoshioka 2021 [70]	Compared numerical data and percentage and used *p* values. OR Shapiro–Wilk test	TB before bed Sometimes/no Always	Participants who brushed their teeth before bedtime every night had lower BMI and LDL-cholesterol than those who did not brush nightly (*p* =< 0.05).
Zhang 2023 [75]	OR and aOR; survey-weighted descriptive statistics; Chi-squared tests; Multivariable logistic regression models. Multivariable linear models. Multicollinearity between the covariates.	Flossing Yes No	The prevalence of poor glycemic control was significantly lower among flossers than non-flossers (29.1% vs. 39.6%, respectively); *p* < 0.01. Flossers were 39% less likely than non-flossers to have periodontitis aOR 0.61 [ 95%CI, 0.43–0.88]. Flossing was significantly associated with a HbA1c reading that was 0.3% lower than non-flossers, adjusted for covariates (β = −0.3, 95% CI −0.58, −0.02, *p* = 0.037).
Zhuang 2021 [49]	Compared numerical data, percentage, *p* values, and HR used.	TB frequency Rarely/Never Sometimes/Always Frequency of gum bleeding Rarely Sometimes Always	Compared with frequent TB, the multivariable adjusted HRs for less frequent TB (rarely or never) were HR 1.28 (95% CI, 1.21, 1.34) for major vascular deaths, HR 1.23 (95% CI, 1.13, 1.33) for major coronary deaths, HR 1.23 (95% CI, 1.13, 1.33) for IHD caused deaths, HR 1.13 (95% CI, 1.06, 1.1)) for cancer caused deaths, HR 1.42 (95% CI, 1.23, 1.6)) for COPD caused deaths, and HR 1.49 (95% CI, 1.05, 1.28) for liver cirrhosis. TB behavior was not associated with death from T2DM and CKD. Compared with those who brushed teeth regularly, those who never or rarely brushed teeth had higher risk of MVE HR 1.12 (95% CI, 1.09, 1.15), stroke HR 1.08 (1.05–1.12), intracerebral hemorrhage HR 1.18 (1.11–1.26), and pulmonary heart disease HR 1.22 (1.13–1.32). No significant results were observed for subarachnoid hemorrhage HR 0.97, 0.74–1.26) and IHD HR 1.00 (0.97–1.04). Those who did not brush teeth also had increased risk of cancer HR 1.09 (1.04–1.14), COPD HR 1.12 (1.05–1.20), liver cirrhosis HR 1.25 (1.09–1.44), and all-cause death HR 1.25 (1.21–1.28), but not T2DM HR 0.94 (0.86–1.03) and CKD HR 0.98 (0.81–1.18).
Zhou 2024 [60]	Mean/SD, One-way analysis of variance, Pearson χ test, Kaplan–Meier survival curves, HR, 95% CI, Cox proportional hazards	TB frequency <1× daily 1× daily ≥2× daily	Compared to TB ≥ 2× daily, participants with lower toothbrushing frequency had higher risks of the following: All-cause mortality: 1× daily HR = 1.16; (95% CI = 1.10, 1.22) *p* < 0.001 >1× daily HR = 1.27, (95% CI = 1.00, 1.61) *p* < 0.048 CVD mortality: 1× daily HR = 1.12; (95% CI = 1.12, 1.34) >1× daily HR = 1.54, (95% CI = 1.09, 2.17) Stroke mortality: 1× daily HR = 1.47; (95% CI = 1.28, 1.70) >1× daily HR = 1.68; (95% CI = 0.96, 2.92) Respiratory disease mortality: (*p* for trend = 0.022): 1× daily aHR = 1.18; (95% CI = 1.01, 1.37) >1× daily aHR = 1.44; (95% CI = 0.81, 2.57) No associations were found for IHD and cancer mortality.

*: note; %: percentage; ACS: acute coronary syndrome; aHR: adjusted hazard ratio; aOR: adjusted odds ratio; ASCVD: atherosclerotic cardiovascular disease; BMI: body mass index; BP: blood pressure; CAD: coronary artery disease; CHD; coronary heart disease; CHF: congestive heart failure; CI: confidence interval; CKD: chronic kidney disease; CPITN/CPI: Community Periodontal Index of Treatment Needs; CRP: C-reactive protein; CVD: cardiovascular disease; DM: diabetes mellitus; DL: dyslipidaemia; DM: diabetes mellitus; DMFT—decayed missing and filled teeth; FPG: fasting plasma glucose; GCF: gingival crevicular fluid; HC: health coaching; HDL: high-density lipoprotein; HE: health education; HR: hazard ratio; hs-CRP high-sensitive c-reactive protein; HUA: hyperuricemia; IHD; ischemic heart disease: IL-1B; cytokine interleukin-1β; IRR: incidence risk ratios; LDL: low-density lipoprotein; MACE: major adverse cardiac events; MI: myocardial infarction; MVE: major vascular event; OHS: oral hygiene self-care; OR: odds ratios; QoL: quality of life; RCT: randomized controlled trial; RR: risk ratio; SD: standard deviation; SLZ: salivary lysosome; STL: significant tooth loss; T2DM: type II diabetes mellitus; TIA: transient ischemic attack; tPAI-1: tissue plasminogen activator inhibitor 1; WBC: white blood cell count.

**Table 3:** Quality assessment of included non-RCT studies using NOS.

First Author, Year	S1	S2	S3	S4	C1	O1	O2	O3	Overall Score & Assessed Quality
Afsar 2013 [80]	★		★	★	★★	★	★	★	8—Good
Aggarwal 2012 [34]	★				★		★	★	4—Poor
Almas 2003 [35]		★	★★	★			★	★	6—Poor
Assante 2020 [33]	★		★	★	★	★		★	6—Good
Chang 2020 [37]	★	★	★		★★	★	★	★	8—Good
Chang 2020 [32]	★		★			★	★	★	5—Fair
Chang 2021 [36]	★	★	★		★★	★	★		6—Good
Chang 2021 [58]	★	★	★	★	★★	★	★	★	9—Good
Cho 2021 [62]	★	★	★	★	★★	★	★	★	9—Good
Choi 2019 [51]			★	★	★★	★	★	★	7—Fair
Cinar 2013 [79]	★		★		★	★		★	5—Poor
Cinar 2015 [64]		★				★	★		3—Poor
deOliveira 2010 [65]	★	★	★		★	★	★	★	7—Good
deSouza 2014 [38]	★	★	★	★	★	★	★	★	8—Good
Frisbee 2010 [66]	★	★	★	★	★★	★	★	★	9—Good
Fujita 2009 [67]	★		★	★	★	★	★		7—Good
Guo 2023 [53]			★★	★	★★	★	★	★	8—Good
Han 2021 [72]	★	★	★	★	★★	★	★	★	9—Good
Hiramatsu 2022 [81]	★	★	★	★		★	★	★	7—Fair
Hirano 2022 [39]	★		★	★	★★	★	★		7—Good
Huang 2023 [71]	★	★	★	★	★★	★	★	★	8—Good
Huh 2023 [57]	★	★	★	★	★★	★	★	★	9—Good
Hwang 2018 [68]	★		★	★	★★	★		★	7—Good
Hwang 2022 [56]	★	★	★	★	★★	★	★	★	9—Good
Isomura 2023 [54]	★		★★	★	★★	★	★	★	9—Good
Jain 2015 [77]	★	★	★	★	★★	★	★	★	9—Good
Jangam 2017 [51]	★		★			★	★	★	5—Poor
Janket 2023 [76]	★	★	★	★	★★	★	★	★	9—Good
Joshipura 2017 [41]	★	★	★★	★	★★	★	★		9—Good
Joshipura 2020 [42]	★	★	★★	★	★★	★	★	★	10—Good
Kim 2022 [52]	★		★	★	★★	★	★	★	8—Good
Kobayashi 2020 [40]	★	★	★	★	★★	★	★		8—Good
Kuwabara 2016 [69]	★		★		★★	★	★	★	7—Fair
Kuwabara 2017 [43]	★	★	★	★	★★	★	★	★	9—Good
Long 2023 [59]	★	★	★		★★	★	★	★	8—Good
Luo 2022 [30]	★	★	★		★★	★	★	★	8—Good
Matsui 2022 [55]	★	★	★★	★	★★	★	★	★	10—Good
Moon 2024 [73]	★	★	★	★	★★	★	★	★	9—Good
Palmer 2015 [45]	★		★	★	★	★	★		6—Fair
Park 2019 [44]	★	★	★	★	★★	★	★		8—Good
Patel 2021 [78]	★	★	★		★	★	★	★	7—Good
Reichert 2015 [46]			★★	★	★★	★		★	7—Good
Song 2021 [47]	★		★		★★	★	★		6—Fair
Song 2021 [48]	★		★		★★	★	★	★	7—Fair
VanWormer 2013 [74]	★	★	★	★	★★	★	★		8-Good
Vogtmann 2017 [61]	★	★	★★	★	★★	★	★	★	10—Good
Wang 2022 [31]	★	★	★	★	★★	★	★	★	9—Good
Winning 2017 [50]	★	★	★★	★	★	★	★	★	9—Good
Yoshioka 2021 [70]			★		★★	★	★	★	6—Fair
Zhang 2023 [75]	★	★	★		★★	★	★		7—Good
Zhuang 2021 [49]	★		★	★	★★	★	★		7—Good
Zhou 2024 [60]	★	★	★	★	★★	★	★	★	9—Good

Selection: (S1) Representativeness of the exposed cohort. (S2) Selection of the non-exposed cohort. (S3) Ascertainment of exposure. (S4) Demonstration that outcome of interest was not present at start of study. Comparability: (C1) Comparability of cohorts on the basis of the design or analysis controlled for confounders. Outcome: (O1) Assessment of outcome. (O2) Was follow-up long enough for outcomes to occur. (O3) Adequacy of follow-up of cohort. NOS tool included selection, outcome, and comparability sections, and scores were categorized as follows: high quality/low risk of bias (7–9 stars), moderate quality/medium risk of bias (4–6 stars), and low quality/high risk of bias (0–3 stars) [74].

## Data Availability

No new data were created for this review.

## Supplementary Materials

The following supporting information can be downloaded at: https://www.mdpi.com/article/10.3390/ijerph21101319/s1.
