# Supplementary material for: Impact of Oral Hygiene Practices in Reducing Cardiometabolic Risk, Incidence, and Mortality: A Systematic Review
Source: Int J Environ Res Public Health. 2024 Oct 4;21(10):1319. doi: 10.3390/ijerph21101319 (PMC11508105; doi:10.3390/ijerph21101319)
Supplement: Supplementary file 1 [file ijerph-21-01319-s001.zip › ijerph-3225026-supplementary.pdf]

Supplementary Material S1. Database search strategies.

MEDLINE Search

Database: Ovid MEDLINE(R) ALL <1946 to July 23, 2021>
Search Strategy:
----

1 Oral Hygiene/ (13271)
2 oral hygiene*.mp. (22671)
3 dental hygiene*.mp. (3209)
4 Toothbrushing/ (7817)
5 tooth?brushing.mp. (8806)
6 interdental clean*.mp. (216)
7 flossing.mp. (779)
8 interdental brush*.mp. (168)
9 water flossing.mp. (4)
10 Dental Devices, Home Care/ (2031)
11 air floss.mp. (4)
12 (Oxyjet or Waterpik or "Water Pik" or "Oral Breeze" or PowerFloss or "Hydro Floss" or "Water Jet" or Aquajet or Interplak or h2ofloss or "Perio Pik" or "Pik Pocket" or Pickpocket* or Softpick or Softpik).tw. (832)
13 1 or 2 or 3 or 4 or 5 or 6 or 7 or 8 or 9 or 10 or 11 or 12 (32271)
14 Cardiovascular Diseases/ (157877)
15 cardiovascular disease*.mp. or CVD.tw. (291931)
16 cardiometabolic disease*.mp. or CMD.tw. (5407)
17 Atrial Fibrillation/ (60182)
18 atrial fibrillation*.mp. (90364)
19 Myocardial Infarction/ (169956)
20 myocardial infarction*.mp. (257898)
21 Heart Failure/ (126427)
22 heart failure*.mp. (222695)
23 Coronary Disease/ (131562)
24 coronary heart disease*.mp. (52543)
25 Stroke/ (111036)
26 stroke*.mp. (325820)
27 Cerebrovascular Disorders/ (47068)
28 cerebrovascular accident*.mp. (7499)
29 Angina Pectoris/ (32715)
30 angina*.mp. (73101)
31 14 or 15 or 16 or 17 or 18 or 19 or 20 or 21 or 22 or 23 or 24 or 25 or 26 or 27 or 28 or 29 or 30 (1176203)
32 Diabetes Mellitus, Type 2/ (144006)
33 type 2 diabetes mellitus*.mp. (50689)
34 T2D.tw. (11948)
35 adult onset diabet*.mp. (547)
36 non insulin dependent diabet*.mp. (10078)
37 Hyperglycemia/ (29222)
38 hyperglycaemia*.mp. (10982)
39 32 or 33 or 34 or 35 or 36 or 37 or 38 (197257)
40 exp Renal Insufficiency, Chronic/ (121269)
41 chronic renal insufficien*.mp. (5192)
42 chronic renal failure*.mp. (24150)
43 Diabetic Nephropathies/ (26634)
44 diabetic nephropath*.mp. (34251)
45 Kidney Failure, Chronic/ (96131)
46 chronic kidney failure*.mp. (1516)
47 chronic kidney diseas*.mp. (61119)
48 40 or 41 or 42 or 43 or 44 or 45 or 46 or 47 (189013)
49 13 and 31 (656)
50 13 and 39 (187)
51 13 and 48 (100)
52 31 or 39 or 48 (1492161)
53 13 and 52 (911)

Supplementary Material S1. Database search strategies.

Embase Search

Database: Embase Classic <1947 to 1973>, Embase <1974 to 2021 July 23> Search Strategy:
1 mouth hygiene/ (28031)
Annotation: in ovid comes up as oral hygiene.
2 oral hygiene*.mp. (15640)
3 mouth hygiene*.mp. (28074)
4 dental hygiene*.mp. (3215)
5 tooth brushing/ (11787)
6 tooth?brushing.mp. (2849)
7 Interdental clean*.mp. (210)
8 flossing.mp. (879)
9 interdental brush*.mp. (189)
10 water flossing.mp. (3)
11 dental device/ (3868)
12 air floss.mp. (4)
13 (Oxyjet or Waterpik or "Water Pik" or "Oral Breeze" or PowerFloss or "Hydro Floss" or "Water Jet" or Aquajet or Interplak or h2ofloss or "Perio Pik" or "Pik Pocket" or Pickpocket* or Softpick or Softpik).tw. (1068)
14 1 or 2 or 3 or 4 or 5 or 6 or 7 or 8 or 10 or 11 or 12 or 13 (47465)
15 cardiovascular disease/ (291221)
16 cardiovascular disease*.mp. or CVD.tw. (449975)
17 cardiometabolic disease*.mp. or CMD.tw. (7324)
18 atrial fibrillation/ (74929)
19 atrial fibrillation*.mp. (162724)
20 heart infarction/ (290347)
Annotation: In Ovid comes up as myocardial infarction
21 heart infarction*.mp. (370500)
22 myocardial infarction*.mp. (316956)
23 heart failure/ (265620)
24 heart failure*.mp. (440360)
25 ischemic heart disease/ (136302)
Annotation: In Ovid comes up as coronary disease. In embase - iscaemic heart disease and coronary artery disease.
26 coronary artery disease/ (205880)
27 ischemic heart disease*.mp. (154143)
28 Coronary artery disease*.mp. (252567)
29 cerebrovascular accident/ (230980)
Annotation: In Ovid comes up as Stroke
30 cerebrovascular accident*.mp. (235328)
31 stroke*.mp. (495245)
32 cerebrovascular disease/ (60815)
33 angina pectoris/ (65757)
34 angina*.mp. (121537)
35 15 or 16 or 17 or 18 or 19 or 20 or 21 or 22 or 23 or 24 or 25 or 26 or 27 or 28 or 29 or 30 or 31 or 32 or 33 or 34 (1948340)
36 Type 2 diabetes mellitus/ (279289)
37 type 2 diabetes mellitus*.mp. (75778)
38 T2D.tw. (22640)
39 adult onset diabet*.mp. (760)
40 non insulin dependent diabet*.mp. (281831)
41 hyperglycemia/ (105766)
42 hyperglycaemia*.mp. (18136)
43 36 or 37 or 38 or 39 or 40 or 41 or 42 (382382)
44 chronic kidney failure/ (110372)
Annotation: renal insufficiency in Medline comes up as kidney failure.
45 chronic kidney failure*.mp. (110993)
46 Chronic renal failure*.mp. (34425)
47 chronic renal insufficien*.mp. (7107)
48 Chronic kidney diseas*.mp. (109979)
49 diabetic nephropathy/ (46127)
50 44 or 45 or 46 or 47 or 48 or 49 (229330)
51 14 and 35 (1347)
52 14 and 43 (409)
53 14 and 50 (189)
54 35 or 43 or 50 (2404146)
55 14 and 54 (1831)

Supplementary Material S1. Database search strategies.

CINHAL Search

Each search: Expanders - Apply equivalent subjects Search modes - Boolean/Phrase Interface - EBSCOhost Research Databases Search Screen - Advanced Search Database - CINAHL Complete Display. Tuesday, July 27, 2021 12:46:16AM

S1 (MH "Oral Hygiene+") OR "Oral Hygiene*"
S2 (MH "Dental Hygiene") OR "dental hygiene*"
S3 (MH "Toothbrushing") OR "tooth?brushing*"
S4 TI ((Oxyjet or Waterpik or "Water Pik" or "Oral Breeze" or PowerFloss or "Hydro Floss" or "Water Jet" or Aquajet or Interplak or h2ofloss or "Perio Pik" or "Pik Pocket" or Pickpocket* or Softpick or Softpik)) OR AB ((Oxyjet or Waterpik or "Water Pik" or "Oral Breeze" or PowerFloss or "Hydro Floss" or "Water Jet" or Aquajet or Interplak or h2ofloss or "Perio Pik" or "Pik Pocket" or Pickpocket* or Softpick or Softpik))
S5 (MH "Cardiovascular Diseases+") OR "cardiovascular disease*"
S6 TI (CVD or CMD) OR AB (CVD or CMD)
S7 "interdental clean*"
S8 "flossing*"
S9 "interdental brush*"
S10 "water flossing"
S11 (MH "Dental Devices, Home Care+") OR "Dental Devices, Home Care*"
S12 "air floss*"
S13 "cardiometabolic disease*"
S14 (MH "Atrial Fibrillation") OR "atrial fibrillation*"
S15 (MH "Myocardial Infarction+") OR "myocardial infarction*"
S16 (MH "Heart Failure+") OR "heart failure*"
S17 (MH "Coronary Disease+") OR "coronary heart disease*"
S18 "stroke*" OR (MH "Stroke+")
S19 (MH "Cerebrovascular Disorders+") OR "Cerebrovascular Disorders*"
S20 "cerebrovascular accident*"
S21 (MH "Angina Pectoris+") Expanders - Apply
S22 "angina*" Expanders - Apply equivalent subjects Search modes - Boolean/Phrase
S23 (MH "Diabetes Mellitus, Type 2") OR "type 2 diabetes mellitus*"
S24 TI T2D OR AB T2D
S25 (MH "Diabetes Mellitus, Type 2") OR "adult onset diabet*"
S26 "non insulin dependent diabet*"
S27 (MH "Hyperglycemia+") OR "hyperglycaemia*"
S28 (MH "Renal Insufficiency, Chronic+") OR "chronic renal insufficien*"
S29 (MH "Kidney Failure, Chronic+") OR "chronic renal failure*"
S30 (MH "Diabetic Nephropathies") OR "diabetic nephropath*"
S31 (MH "Kidney Failure, Chronic+") OR "chronic kidney failure*"
S32 "chronic kidney diseas*" Expanders - Apply
S33 S1 OR S2 OR S3 OR S4 OR S7 OR S8 OR S9 OR S10 OR S11 OR S12
S34 S5 OR S6 OR S13 OR S14 OR S15 OR S16 OR S17 OR S18 OR S19 OR S20 OR S21 OR S22
S35 S23 OR S24 OR S25 OR S26 OR S27
S36 S28 OR S29 OR S30 OR S31 OR S32
S37 S33 AND S34
S38 S33 AND S35
S39 S33 AND S36
S40 S34 OR S35 OR S36
S41 S33 AND S40

Scopus Search

(TITLE-ABS-KEY ("oral hygiene*" OR "dental hygiene*" OR "tooth*brushing" OR "interdental clean*" OR flossing OR "interdental brush*" OR "water flossing" OR "homecare dental device*" OR "air floss" OR oxyjet OR waterpik OR "water pik" OR "oral breeze" OR powerfloss OR "hydro floss" OR "water jet" OR aquajet OR interplak OR h2ofloss OR "perio pik" OR "pik pocket" OR pickpocket* OR softpick OR softpik) AND TITLE-ABS-KEY ("cardiovascular disease*" OR cvd OR "cardiometabolic disease*" OR cmd OR "arterial fibrillation*" OR "myocardial infraction*" OR "heart failure*" OR "coronary heat disease*" OR stroke* OR "cerebrovascular accident*" OR "cerebrovascular disorder*" OR angina*) OR TITLE-ABS-KEY ("type 2 diabetes mellitus*" OR t2d OR "adult onset diabet*" OR "non insulin dependent diabet*" OR hyperglycemia*) OR TITLE-ABS-KEY ("chronic renal insufficiency" OR "chronic renal insufficien*" OR "chronic renal failure*" OR "diabetic nephropathies" OR "diabetic nephropath*" OR "chronic kidney failure" OR "chronic kidney diseas*"))
